# Meta-analysis of the Microbial Diversity Cultured in Bioreactors Simulating the Gut Microbiome

**DOI:** 10.1007/s00248-024-02369-0

**Published:** 2024-04-08

**Authors:** David Felipe Garcia Mendez, Siobhon Egan, Julien Wist, Elaine Holmes, Janeth Sanabria

**Affiliations:** 1 Australian National Phenome Centre and Centre for Computational and Systems Medicine, Health Futures Institute, Murdoch University, Harry Perkins Building, Perth, WA 6150 Australia; 2 Environmental Microbiology and Biotechnology Laboratory, Engineering School of Environmental & Natural Resources, Engineering Faculty, Universidad del Valle – Sede Meléndez, 76001 Cali, Colombia; 3 Chemistry Department, Universidad del Valle - Sede Meléndez, 76001 Cali, Colombia

**Keywords:** In vitro gut, Faecal microbiome, Simulation, Diversity, Hill numbers

## Abstract

Understanding the intricate ecological interactions within the gut microbiome and unravelling its impact on human health is a challenging task. Bioreactors are valuable tools that have contributed to our understanding of gut microbial ecology. However, there is a lack of studies describing and comparing the microbial diversity cultivated in these models. This knowledge is crucial for refining current models to reflect the gastrointestinal microbiome accurately. In this study, we analysed the microbial diversity of 1512 samples from 18 studies available in public repositories that employed cultures performed in batches and various bioreactor models to cultivate faecal microbiota. Community structure comparison between samples using t-distributed stochastic neighbour embedding and the Hellinger distance revealed a high variation between projects. The main driver of these differences was the inter-individual variation between the donor faecal inocula. Moreover, there was no overlap in the structure of the microbial communities between studies using the same bioreactor platform. In addition, α-diversity analysis using Hill numbers showed that highly complex bioreactors did not exhibit higher diversities than simpler designs. However, analyses of five projects in which the samples from the faecal inoculum were also provided revealed an amplicon sequence variants enrichment in bioreactors compared to the inoculum. Finally, a comparative analysis of the taxonomy of the families detected in the projects and the GMRepo database revealed bacterial families exclusively found in the bioreactor models. These findings highlight the potential of bioreactors to enrich low-abundance microorganisms from faecal samples, contributing to uncovering the gut microbial “dark matter”.

**Supplementary Information:**

The online version contains supplementary material available at 10.1007/s00248-024-02369-0.

## Introduction

In the last 20 years, multiple researchers have contributed to understanding the impact of the gut microbiome on human health, and the growing interest in this field is reflected in the escalating body of publications. What started as a goal to describe the diversity of the microorganisms in the human gut and other body sites has shifted focus to the more challenging task of studying the mechanisms by which host-microbiota interactions occur [1]. Previous work has addressed the limitations surrounding the mechanistic study of the gut ecosystem. One significant limitation is that some of the regions of the intestinal tract remain mostly inaccessible. While invasive methods such as endoscopy and nasoenteric probes have provided valuable information on the microbial activity and composition of the different sections of the gut [2, 3], these procedures are expensive, require highly trained personnel and specialised equipment, and may face practical and ethical limitations. In addition, this intestinal environment has a very dynamic nature, showing a high intra-individual and inter-individual variation, being influenced by confounding factors such as diet, lifestyle, and medication, among others [4].

Combining high-throughput sequencing with cultivation-independent methods has provided valuable insights into our understanding of the factors that shape gut ecology. It has given access to a large inventory of taxonomic and functional information on the trillions of microorganisms present in the human gut. Notably, it has also raised awareness of the microbial “dark matter”, which includes multiple bacterial and other microbial species still to be cultured and whose biological functions are yet to be understood [5]. Multiple research groups have highlighted the limitations of culture-independent techniques with regard to fully capturing the bacterial diversity in faecal samples. These include the biases introduced by DNA extraction efficiency between taxa, selecting a given set of primers, and variable sequencing depth between samples [6]. In addition, the extrapolation of functions from genomic information remains challenging, with many genes from the microbial dark matter still to be annotated [5, 7]. Culturing and isolating this microbial diversity is still a challenging task, but it is essential not only to aid in the genetic and phenotypic characterisation of these microorganisms but also to facilitate the shift from correlative studies to causative validation of predicted bacterial functions [8]. Promising advances have been made in the cultivation of multiple species previously considered “unculturable” from the gut microbiota, strongly questioning the prevailing belief that much of the gut microbiome cannot be cultured [9, 10]. However, metagenomic approaches suggest that there is still a large microbial diversity that is not included in these culture collections [5, 11].

All these limitations have motivated the development of multiple models for the cultivation and study of the gut microbiota in vitro and in vivo, including gnotobiotic animals, organoids, cell culture models, microbial cultures, and bioreactors [12, 13]. Microbial cultures performed in batches are a cheap and efficient method (i.e. multiple culture conditions can be tested simultaneously) for studying multiple bacteria-bacteria and bacteria-substrate interactions in short-term experiments. On the other hand, bioreactors are more complex devices whose integrated technology allows them to provide an optimum environment for the desired microbial reactions that better mimic some of the environmental conditions in the human body. This environment can be obtained by precisely controlling the operational parameters of the cultures, such as pH, redox potential, and nutrient feed, among others.

Bioreactors are promising tools that can unlock access to the microbial “dark matter”. The precise control of the culture conditions allows the recovery and growth of hard-to-culture microorganisms. A pertinent example is the cultivation of environmental anammox bacteria, which exhibit slow growth rates ranging from 2 days to several weeks. Moreover, their growth is inhibited by the accumulation of their product metabolites in the culture, requiring continuous cultivation methods such as up-flow column reactors [14, 15]. The research teams behind the human gut models have demonstrated that they can culture reproducible bacterial communities with a diversity similar to their initial inoculum. Faeces are commonly used as inoculum, but other inocula have been tested, such as ileostomy effluents, microbial isolates from faeces, and synthetic communities [16–18]. Based on the number of publications derived from these models, the most well-known platforms are SHIME [19], TIM-2 [20], SIMGI [21], PolyFermS [22], ARCOL [23], and the three-stage model from Macfarlane et al. [24]. Although bioreactor technology has made significant progress, there are multiple gaps that limit the potential of these devices to uncover new biology and be fully exploited. For instance, there is still a huge gap in our understanding of the impact of operational parameters such as nutrient medium, retention time, flow and agitation, and pH on the ecology of the microbial communities recovered in bioreactors [25]. Moreover, there is a lack of studies that describe and compare the microbial communities cultured in these devices. This knowledge can be useful for refining current models to reflect the composition and behaviour of the microbiome within the human gastrointestinal tract more accurately. Given the strong inter-individual differences and impact of operational parameters on compositional analysis, comparison of multiple systems and conditions is essential to allow appropriate interpretation of data from bioreactor models. However, the analytical cost and required experimental time prohibit high throughput analysis. Co-analysis of archived sequencing data from multiple studies and research groups has the potential to allow a clearer definition of the limits and appropriate use of bioreactor technology.

In this work, we aimed to describe and compare the taxonomic diversity and structure of the microbial communities cultured in these devices by performing a meta-analysis of the 16S rRNA sequences of over 1512 samples available in the NCBI Sequence Read Archive (SRA). These samples were retrieved from projects that used microbial cultures performed in batches and bioreactors seeded with human faeces. We explored the relationships between sample input and operational parameters in regard to their impact on microbial community structure and investigated differences in microbial communities before and after the inoculation of stool samples in three of the bioreactor models.

## Methods

### Search in the NCBI Sequence Read Archive (SRA)

**Fig. 1:**
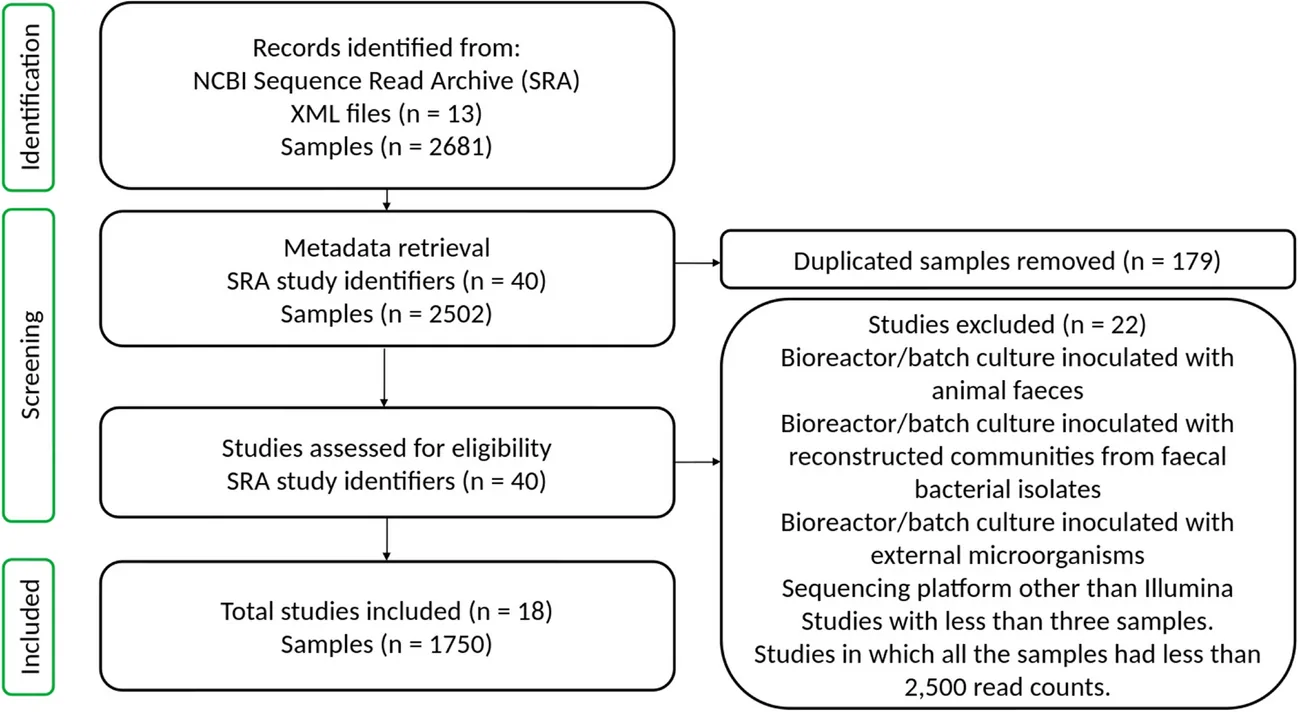
Flow diagram showing the data retrieval process

Our search aimed to retrieve 16S rRNA amplicon sequences from samples obtained from studies that used either (i) bioreactors to simulate the gut environment or (ii) microbial cultures performed in batches, both inoculated with human faeces. For this purpose, we performed multiple searches in the SRA (https://www.ncbi.nlm.nih.gov/sra) using each one of the following keywords: “continuous flow bioreactor model”, “fecal batch culture”, “gut bioreactor”, “gut microbiota cultured in vitro”, “in vitro digestion”, “PolyfermS”, “SHIME”, “simulated gastrointestinal”, “simulation of the colon”, “simulation of the gut”, and “TIM-2”. After each search, we used the SRA Run selector tool to download the metadata of all the samples into an XML file. This search was performed in September 2022. Then, in-house scripts in R were used to remove duplicates and filter the studies and samples based on selection criteria (Fig. 1) (https://zenodo.org/doi/10.5281/zenodo.8045625—file:1_Metadata_retrieve.Rmd). The accession numbers of the projects were used to retrieve the FTP address of the samples using ffq [26], and their sequences were downloaded from the NCBI. Additional metadata, such as the operational conditions of the bioreactor and composition of the culture media, were obtained from the published papers of the projects to support further analyses. During the search, a special focus was given to projects in which the composition of the faecal inoculum could be identified, allowing comparisons with the samples obtained from the bioreactors.

### Informatic Analysis

Data visualisation was performed using R statistical software (v 4.1.2, R Core Team 2022) in RStudio (v2022.12.0, Posit Team 2022). High-throughput sequence data were analysed using *dada2* (v 1.16) [27]. First, we analysed the samples from the selected projects individually and manually adjusted the input parameters of the dada2 package to maximise the number of reads that passed the denoising and quality filtering steps, while reducing the number of total errors in the reads. The parameters used to set up the dada2 algorithms are summarised in Additional file 1: Table S1. Then, sequences were de-replicated, and high-resolution amplicon sequence variants (ASVs) were produced, followed by the removal of chimaeras. Taxonomy was assigned to ASVs using the BLASTN algorithm with NCBI RefSeq 16S rRNA database as the reference (downloaded June 2022—available in Zenodo: https://zenodo.org/doi/10.5281/zenodo.8045625—file:/supporting_metadata/bacteria.16SrRNA.zip). Briefly, we filtered and selected the top hit with the highest percentage of identical positions (pident) for each ASV. The subject sequence id (sseqid) obtained from BLAST was used to search the NCBI unique record identifier (UID) using the package taxize (v 0.9.100). Taxonomic lineage was retrieved using the function classification from the package *taxize* (code available in Zenodo: https://zenodo.org/doi/10.5281/zenodo.8045625—file:Taxonomy.Rmd). Due to the region and length variation of the 16S rRNA sequences obtained from the various studies and limitations in accurate taxonomic assignment to genus and species level for short-read 16S rRNA data, all taxonomy was collapsed to the family level to enable meaningful comparison. Data were analysed using *phyloseq* (v 1.38) and other R packages (code available in Zenodo: https://zenodo.org/doi/10.5281/zenodo.8045625).

### Comparison of the Community Structure Between Samples (β-Diversity) and Within Samples (α-Diversity)

Samples not directly obtained from the bioreactor (e.g. faecal samples), those with zero sequences after bioinformatic analysis, and those with less than 2500 read counts were removed from the data set used for diversity analysis. Beta diversity was assessed using principal component analysis (PCA) and with the non-linear dimension reduction algorithms, t-distributed stochastic neighbour embedding (t-SNE), and Uniform Manifold Approximation and Projection (UMAP) with packages *Rtsne* (v 0.16) and *umap* (v 0.2.9). Hellinger distances were used for β-diversity analysis to account for large differences in sequencing depth while avoiding rarefying of samples. The limitations of rarefying microbiome data have been addressed previously [28]. The Hellinger distances were calculated by applying the Hellinger transformation to the compositional data matrix and then computing the Euclidean distance among samples. For α-diversity analyses, a series of Hill numbers were calculated for each sample at diversity orders *q* = 0–3 using the package *hilldiv* (v 1.5.1). Subsequently, these variables were plotted to obtain a diversity profile of each study. In addition, traditional diversity indices such as Observed, Chao1, Shannon, and inverse Simpson indices were calculated using packages *phyloseq* (v 1.38) and *ggstatsplot* (v 0.11.0). Statistical analysis of α-diversity measures was performed using non-parametric tests with the holm p-adjustment method, Kruskal–Wallis one-way ANOVA (no. groups > 2), or Mann–Whitney *U* test (no. groups = 2). Pair-wise comparisons for paired combinations of experimental parameters (e.g. single versus pooled donor) were produced for ANOVA analysis using Dunn’s non-parametric all-pairs comparison.

### Analysis of Amplicon Sequence Variants (ASVs) Enrichment in the Bioreactors

We explored the metadata of the studies available in the NCBI to select the ones that allowed assessment of (i) sequences of the initial faecal inoculum; (ii) source donor; (iii) if it was a multistage design, the gut compartment that was simulated; and (iv) if it included a mucosal component, the lumenal or mucosal origin of the sample. By mucosal component, we refer to bioreactors in which support media such as plastic carriers covered with mucin were introduced to allow biofilm growth, whereas lumenal sample refers to samples taken from the unstructured culture media. Based on these criteria, five projects were further selected to evaluate the enrichment of ASVs in the bioreactors. “ASVs enrichment” in this study is defined as the ASVs not present in the initial faecal sample (inoculum), as determined from the sequencing data analysis that appeared after the cultivation of the microbial community in the bioreactor. *In-house* scripts in R were used to filter the ASVs absent in the initial inoculum (number of reads = 0) but that were present in the bioreactors (number of reads > 0). For studies with multiple donors, the biodiversity comparison was made between samples from the same donor. On the other hand, if the study included replicates, only ASVs absent in both faecal sample replicates and present in both bioreactor sample replicates were selected. Finally, the taxonomic classification of these ASVs was obtained from the previous identification performed with BLAST (see “Informatic Analysis” for details). A table describing selected samples is available in Additional file 1: Table S5.

### Comparison of the Taxonomy Between Samples and the Data Repository for Human Gut Microbiota (GMrepo)

The overlap of taxonomy at the family level found in the vitro models and that previously documented by direct sequencing of the human faecal microbiome was explored. Firstly, a list of all the species available in the Data Repository for Human Gut Microbiota (GMrepo) [29] was downloaded (February 2024—available in Zenodo: https://zenodo.org/doi/10.5281/zenodo.8045625—file:/supportingmetadata/GMREPO). Then, the package *taxize* was used to retrieve the taxonomic lineage by searching for their NCBI taxon ID. Finally, the taxa lists obtained from the projects and the samples in GMrepo were compared using the function *setdiff* in R.

### Data and Code Availability

No new sequencing datasets were generated during the current study. Details of accession numbers for data analysed can be found in Additional file 1: Table S2. Original R scripts and metadata files are available in Zenodo ( https://zenodo.org/doi/10.5281/zenodo.8045625).

## Results

### Search in the NCBI Sequence Read Archive (SRA)

A total of 18 studies were selected after the search in the SRA, accounting for 1750 samples from all the projects (Additional file 1: Table S2). Subsequently, samples directly from faeces (*n* = 79), those with zero reads after bioinformatic processing (*n* = 63), and those with less than 2500 read counts (*n* = 96) were removed, leaving the final number of samples as 1512. The library size of selected samples was between 2661 and 419,940 reads (Additional file 1: Fig. S1). Moreover, the rarefaction curves obtained from plotting the number of observed OTUs against the sequencing depth (number of reads) in the samples showed that most cultures reached a plateau, suggesting that the sequencing depth was adequate (Additional file 1: Fig. S2).

The selected projects included samples from five studies that used cultures performed in batch and seven bioreactors with specific configurations: single-stage model (single_stage_Xu_et_al_2019), SHIME, SIMGI, TIM-2, PolyFermS, and ARCOL. These configurations are briefly described in Additional file 1: Table S2. Most of these studies (13/18) targeted the V3-V4 region of the 16S rRNA gene, followed by the V4 region (3/18), and only two studies used each of the V1 or V3 regions, respectively. The metadata on the operational parameters used in these models, including country, inoculum type, pH, and composition of the culture media, were extracted from the papers and are available in Additional file 2.

### Comparison of the Microbial Diversity Between Bioreactor Studies (β-Diversity)

**Fig. 2:**
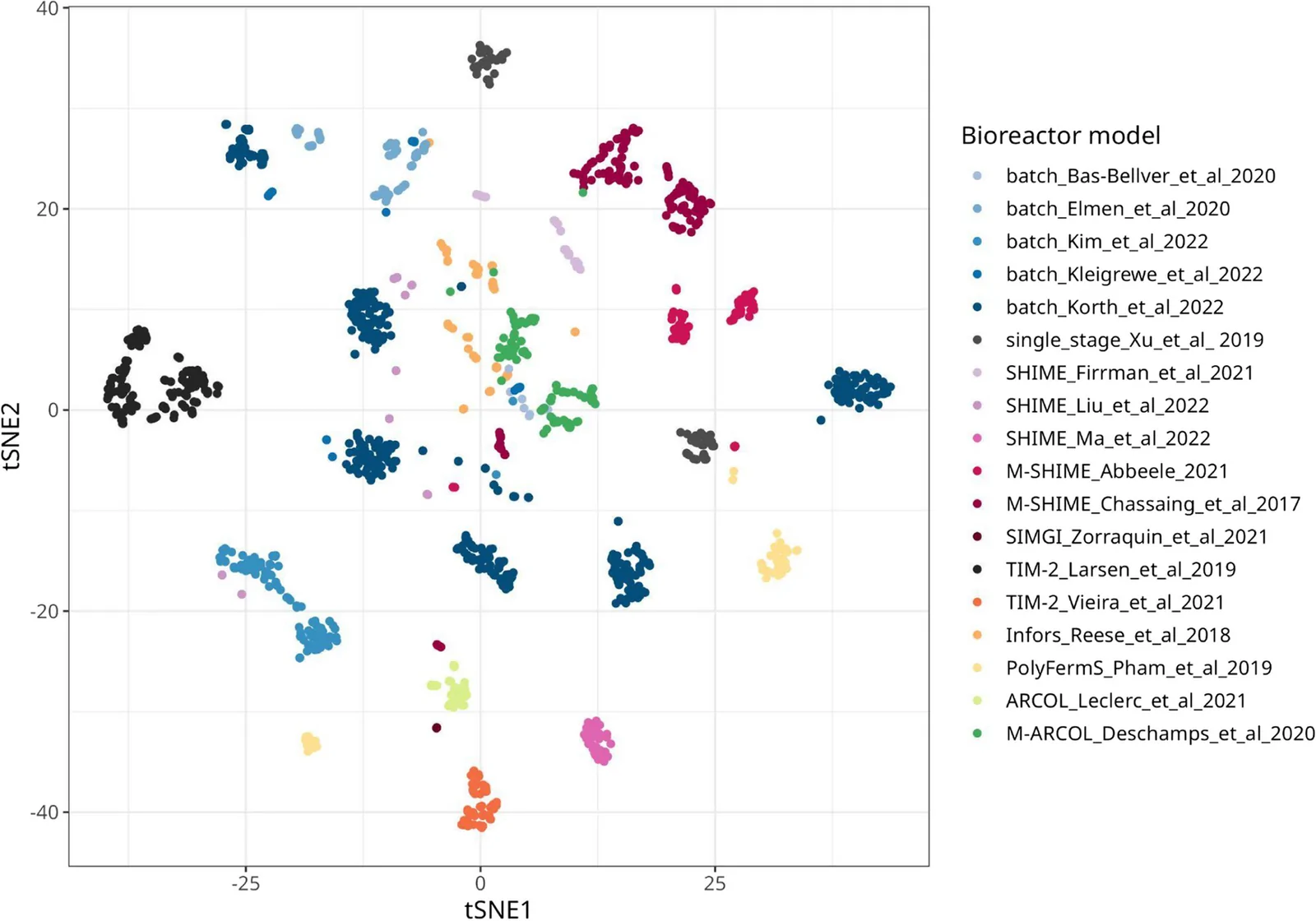
Comparison of microbiome community structure from 18 different studies with 1512 samples. Multi-dimensional scaling plot using t-distributed stochastic neighbour embedding (t-SNE) with Hellinger distance measure of compositional data

The t-SNE analysis grouped the samples by study, with no overlap between samples from the same bioreactor model (Fig. 2). For instance, the samples from SHIME studies were not clustered and remained separated in the t-SNE plot. However, microbial communities from different studies did overlap, except for the ones in SHIME_Ma_et_al_2022, which was notably different from that observed in other studies, as indicated by its remote mapping space (Fig. 2). One of the limitations of using t-SNE for β-diversity analysis is that it preserves the local distances but not the global distances. To overcome this limitation and support our observations, we also performed a PCA over the Hellinger-transformed compositional data. The PCA showed a similar pattern to that observed in the t-SNE (Additional file 1: Fig. S4), indicating that the observations made from the t-SNE are robust. In addition, where studies included more than one donor, the individual donors did not necessarily map closely, indicating the greater impact of the donor over the in vitro model platform. For example, batch_Korth_et_al_2022 screened individual stool samples from four adult volunteers, showing high dispersion in the t-SNE plot (Fig. 2) and PCA (Additional file 1: Fig. S4) based on inter-individual compositional differences. UMAP analysis confirmed the grouping patterns observed by t-SNE but overestimated sample similarity, as reflected by the tightness of the clusters relating to each study (Fig. S3). Importantly, microbial community differences were not driven by variation in the sequenced 16S rRNA gene region once taxonomy was collapsed to the family level (Additional file 1: Fig. S4). Based on visual assessment, operational parameters, such as the carbon-to-nitrogen ratio, sample origin, use of vitamins, number of bioreactor stages, gut compartment, and mucin amount, did not exhibit strong patterns across the dataset (Additional file 1: Fig. S5-6).

### Mean Species Diversity in a Site at a Local Scale (α-Diversity)

**Fig. 3:**
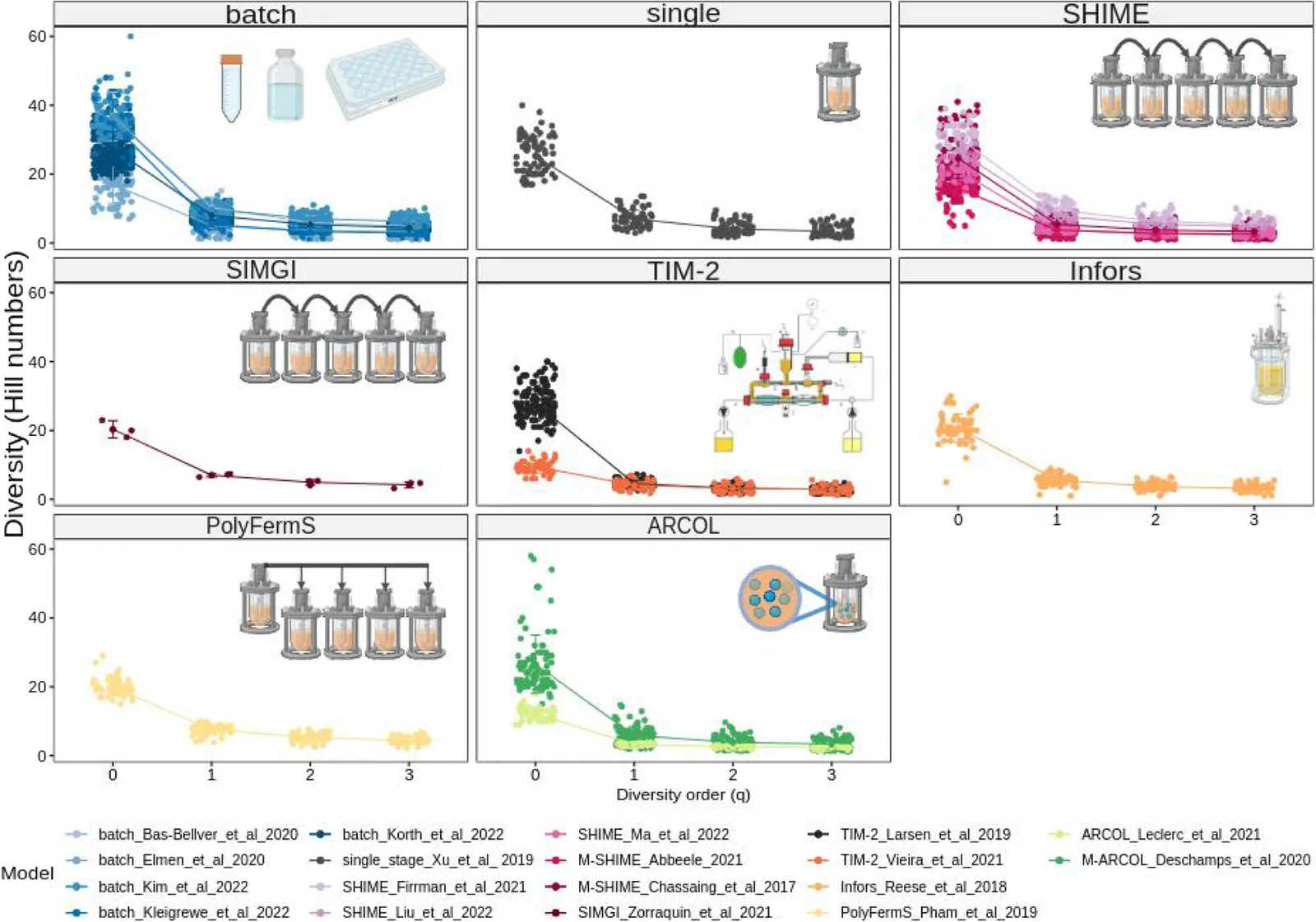
Diversity profiles of the 18 selected studies determined by Hill numbers at diversity orders *q* = 0–3. Diversity profile plots are grouped by bioreactor model type to aid data visualisation and comparison. The sensitivity towards abundant and rare families is modulated using the diversity order “q”. The larger the *q* value, the higher the importance attributed to abundant families. At diversity order *q* = 0, the Hill numbers represent the total number of families in the samples (richness). A value of *q* = 1 weights families by their proportion without disproportionately favouring rare or abundant families. This value yields the exponential of the Shannon index. At *q* = 2, abundant families are over-weighted, and the number yields the multiplicative inverse of the Simpson index. Vertical bars show the standard deviation. This summarised explanation was adapted from Alberdi and Gilbert et al. 2019 [30]

Based on the α-diversity profile determined by Hill numbers, the studies that showed the highest diversity of families were batch_Bas-Bellver_et_al_2020, batch_Kim_et_al_2022, and batch_Kleigrewe_et_al_2022. As can be noted, all these projects used cultures performed in batches (Fig. 3, Additional file 1: Fig. S7). In contrast, the studies that showed the lowest diversity across the profile were ARCOL_Leclerc_et_al_2021 and TIM-2_Vieira_et_al_2021. In addition, samples from bioreactors that used formula-fed infants as an inoculum showed significantly higher Shannon diversity indices when compared to other inocula (Additional file 1: Fig. S8c).

### Comparison of the Taxonomy Between Models and the Faecal Microbiome

**Fig. 4:**
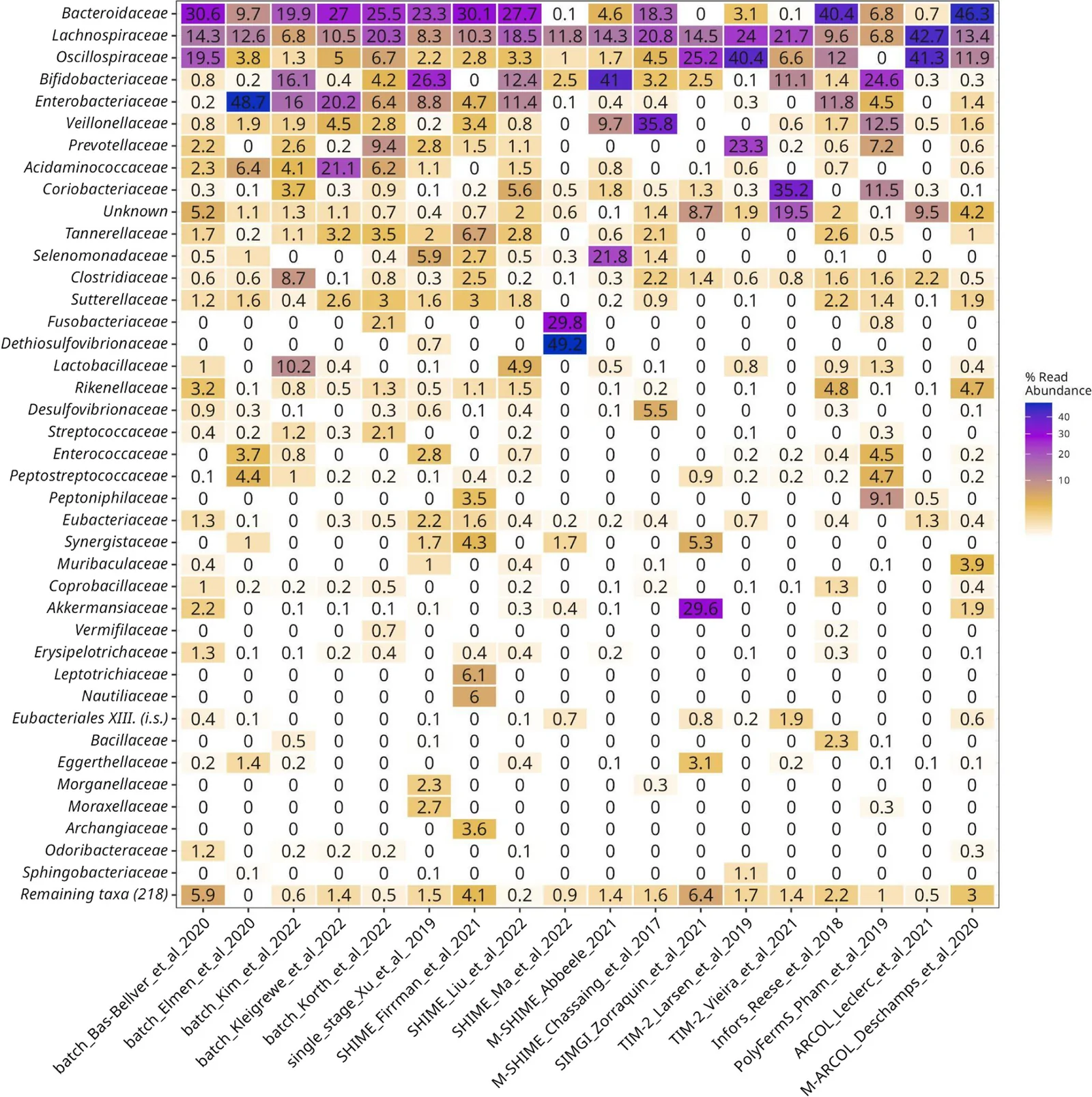
Heatmap of top 40 bacteria families identified in samples (remaining taxa grouped together in bottom row). Compositional data are represented as the total proportion of sequences from all samples in the project (*n* = 1512)

An evaluation of the effect of the different operational parameters and settings of the bioreactor models showed that, as expected, the most abundant groups at the phylum level in the samples were *Bacillota*, *Pseudomonadota*, *Actinomycetota*, and *Bacteroidota* (Additional file 1: Fig. S9). In particular, the study SHIME_Ma_et_al_2022 showed enrichment in *Synergistota* (50.9%) and *Fusobacteriota* (29.8%). In the current meta-analysis, at the family level, the top 5 families that accounted for up to 50% of the abundance in the samples were *Bacteroidaceae*, *Lachnospiraceae*, *Oscillospiraceae*, *Bifidobacteriaceae*, and *Enterobacteriaceae* (Fig. 4).

To focus on the less abundant taxa, we compared the taxonomy of the enriched families in the projects selected for the ASVs enrichment analysis (Additional file 1: Table S6): a family was considered to be enriched based on two criteria: (i) when it was not detected in the faecal inoculum; in other words, there were no ASVs associated to this family in the inoculum and (ii) when we detected at least one read from an ASV belonging to this family in the samples obtained from the bioreactors. The most common enriched families detected across all the samples were *Enterobacteriaceae* (11/36), *Desulfohalobiaceae* (10/36), and *Synergistaceae* (9/36).

Subsequently, we explored whether there was any difference between the list of families observed in the bioreactors and the list of faecal taxa obtained from GMRepo. We found 36 families in the bioreactors and batch cultures that have not been detected in the curated projects of GMRepo; the most prevalent ones were the families *Gracilibacteraceae* (13/18), *Muribaculaceae* (11/18), *Defluviitaleaceae* (9/18), and *Vallitaleaceae* (8/18). However, these families were mostly observed in low relative abundances (< 0.0001%) and occasionally in higher proportions (> 0.1%).

**Fig. 5:**
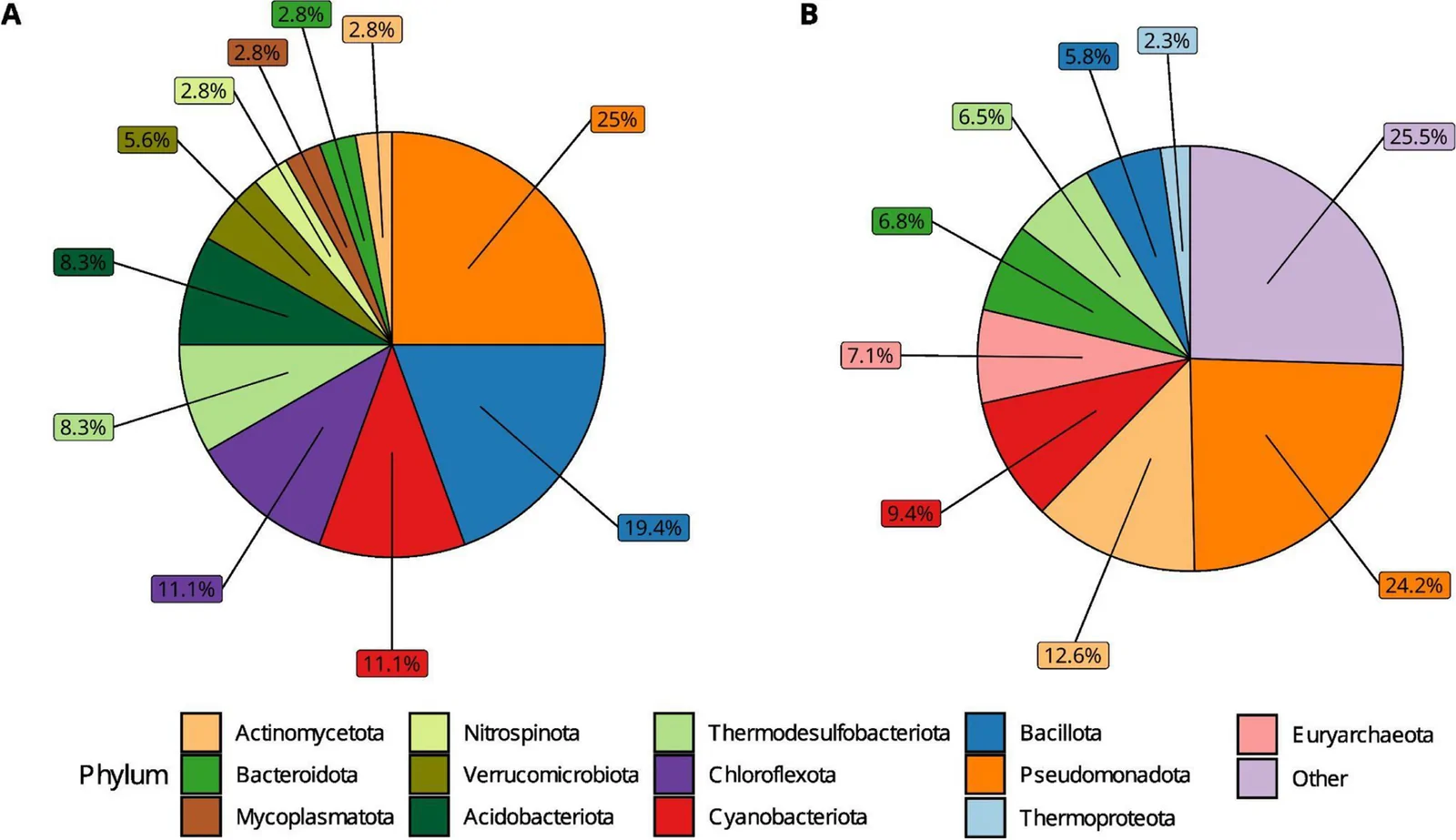
Composition at the phylum level of unique families observed in selected studies and GMRepo. (**A**) Phyla of the families only present in the selected studies. (**B**) Phyla of the families only present in GMRepo. The percentages were obtained by dividing the number of families within each phylum by the total number of families in each category, which are 36 and 310, respectively

At the phylum level, most of these enriched families belonged to *Pseudomonadota* (25.8%), *Bacillota* (25.8%), *Cyanobacteriota* (11.1%), and *Chloroflexota* (12.9%) (Fig. 5A). In addition, the phylum *Nitrospinota* was only detected in the bioreactors. On the other hand, 310 families found in the faecal samples of the GMRepo database were not detected in any of the studies included in the current meta-analysis. Similarly, these families that are distinct only to the GMRepo database are not highly abundant in faecal samples. Most of them belonged to the phyla *Pseudomonadota* (24.2%), *Actinomycetota* (12.6%), and *Cyanobacteriota* (9.4%) (Fig. 5B).

### Analysis of ASVs Enrichment in the Bioreactors

**Fig. 6:**
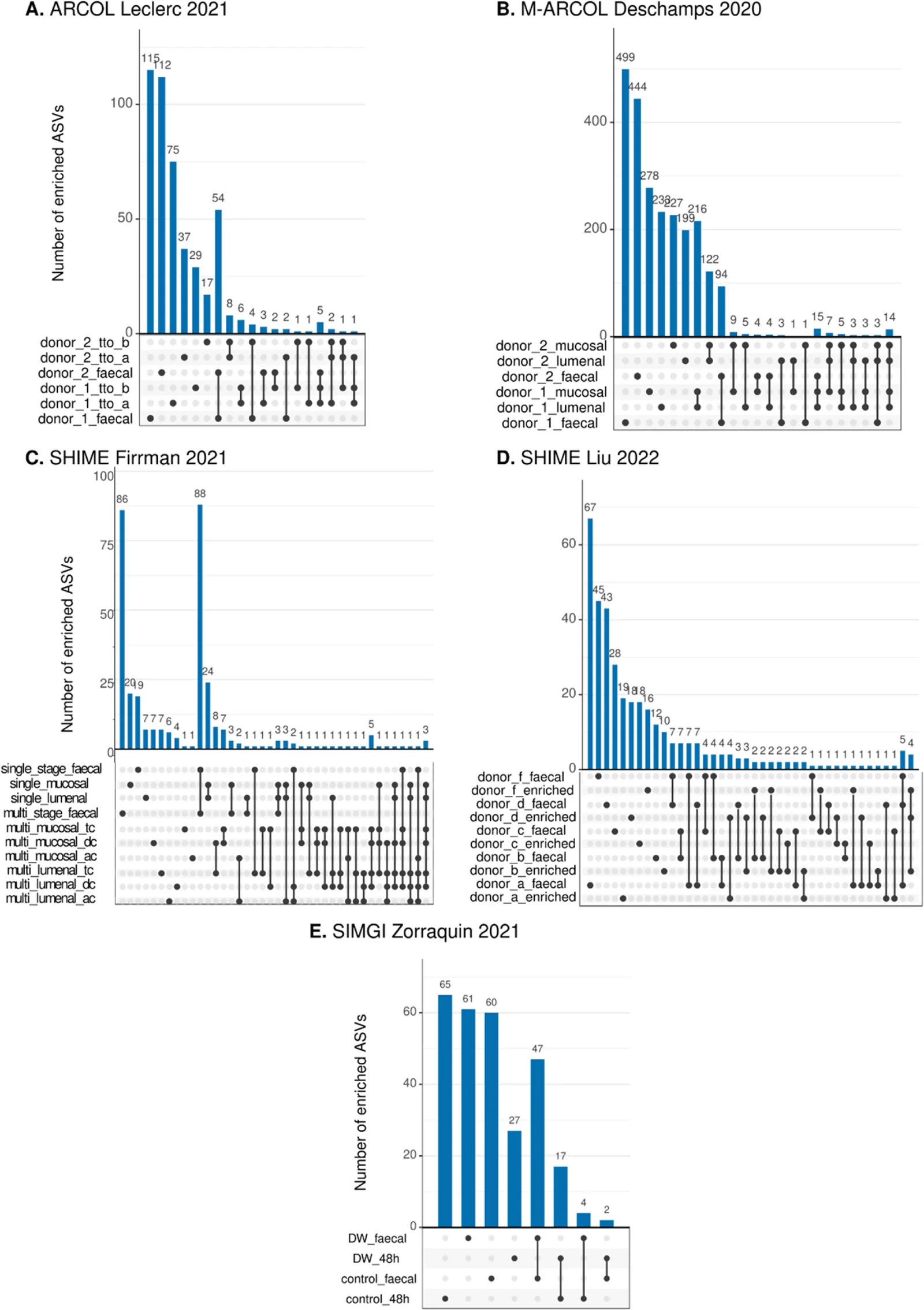
Enrichment analysis of ASVs for selected samples. This graph shows the number of ASVs enriched in five bioreactor models. **A** SHIME_Firrman_et_al_2021. **B** SIMGI_Zorraquin_et_al_2021. **C** ARCOL_Leclerc_et_al_2021. **D** M-ARCOL_Deschamps_et_al_2020. **E** SHIME_Liu_et_al_2022. The number of ASVs in the faecal samples is plotted as a reference (n reads > 0). The samples from the original faecal inoculum are labelled as “_faecal”, while other labels correspond to samples from the bioreactors. The vertical bars represent the number of unique ASVs in each category. The dots and lines represent the comparisons that were performed between samples. Single dots indicate ASVs enriched in a given sample only, while dots connected with a line indicate that ASVs are shared between samples. This plot shows that new ASVs are detected in the bioreactors compared to the faecal inoculum

In our analysis, we found that despite the dominance of a few families in the communities developed in the bioreactors, new ASVs appeared in all the selected models when compared to the inoculum (Fig. 6). Moreover, the results show that modification of the bioreactor design such as adding plastic carriers for promoting biofilm growth increased the number of ASVs detected. For instance, the study of M-ARCOL_Deschamps_et_al_2020 showed 499 ASVs in the faeces of one of the donors. After inoculation in the bioreactor, 227 and 238 new ASVs were detected in the lumenal and mucosal compartments, respectively. A similar trend was observed in the study of SHIME_Firrman_2021.

## Discussion

### Inter-individual Variation Is the Main Driver of the Difference Between Projects

Our results suggest that inter-individual variation in faecal inoculum diversity is the main driver of the difference in family-level composition between studies. The variability among individuals seems to be greater than the selective pressure exerted by the bioreactor model. However, further research is required to validate this claim. An interesting approach would be to culture faecal samples from the same donors in all these bioreactors to determine how the microbial communities are shaped by the culture conditions across different platforms. Furthermore, these findings show how pooling samples to increase the diversity of the inocula comes at the cost of losing information about the community structure of individual samples. Similarly, Isenring et al. [31] suggested that the pooling of samples completely removes inter-individual differences, leading to an artificial community with unpredictable competition and artificial balance among taxa. Moreover, each species that establishes itself in a microbiome plays an important role. Although we cannot observe this importance as a direct metabolic consequence, it may impact both the ecological structure of the community and the downstream microbial metabolites generated by the community as a coherent entity. This information is lost in a pooled sample since the combined microbiota represents multiple independent ecologies. Researchers must carefully consider this effect when selecting the inoculum used in their in vitro models.

### Complex Bioreactor Designs Did Not Achieve a Higher Microbial Diversity

Hill numbers have been proposed as a general, robust, and flexible statistical framework [30], offering multiple advantages over traditional diversity indices such as richness, Shannon, and Simpson [30, 32, 33]. For instance, the interpretation of the Hill measure and its measurement unit is always the same, and the *q* parameter can modulate the sensitivity towards abundant and rare taxa. The steep slope of the profiles in Fig. 3 indicates a high unevenness of the community structure inside the bioreactors, with strong variation in abundance between the represented families. In other words, a few highly abundant families dominate these communities, a common feature of the microbiome of different body sites, such as the vagina and the gut environment. Nevertheless, low-abundance families can still fulfil crucial biological functions, such as those residing in specialised environments like microvilli crypts. If the role of these less common family members is neglected, crucial insights into the community’s overall functioning are lost. For instance, methanogenic bacteria in the intestine and anammox in aquatic environments are challenging to cultivate and often go undetected in the direct sequencing of samples. These facts do not remove their impact, as methanogens influence fermentation, and anammox affects the entire nitrogen cycle.

Moreover, the results suggest that the diversity of the original faecal inoculum greatly impacts the diversity recovered in the bioreactor. However, this analysis was limited because the available metadata did not allow us to identify the samples of the faecal inocula in most of the studies.

Consequently, we could not compare the faecal inocula with the bioreactor’s communities. Despite this limitation, we did find that microbiome communities originating from individuals had a higher α-diversity than those where pooled samples were analysed separately (Mann–Whitney *U* test on Shannon diversity indices, *p* < 0.005. Additional file 1: Fig. S7, Table S4). These results contrast those obtained by Średnicka et al. (2023), who found higher α-diversity metrics in pooled samples from healthy donors than in individual stool samples [34].

In addition, it is worth highlighting the low total number of reads (less than 10,000) observed in multiple samples from the projects TIM-2_Vieira_et_al_2021, ARCOL_Leclerc_et_al_2021, SIMGI_Zorraquin_et_al_2021, and others (Additional File 1: Fig. S1). This low number of reads is below an appropriate minimum to obtain reliable estimates of diversity statistics from these samples. A low number of reads might be obtained due to errors during sample DNA extraction and sequencing. However, an alternative hypothesis is that the operating conditions in those bioreactors might have impacted the number of reads observed in the samples. For instance, a total low microbial biomass in the system can decrease the DNA yield in the sample during extraction, affecting all the subsequent analytical steps.

### Bioreactors as Tools for Unlocking the Microbial Dark Matter

The cultivation of all the microorganisms present in the gut is a challenging task. Although promising advancements have been made in this regard [9, 10], a huge diversity of microorganisms is still to be cultured. This is supported by metagenomic studies that show a huge taxonomic diversity that is not included in culture collections [5, 11]. Moreover, culturomic approaches have demonstrated the distinct benefits of using different culture strategies to analyse the same sample to evidence uncultured species. When multiple plating or enrichment methods are used, a *hidden* diversity appears, extending the number of species that can be detected compared to traditional culture methods. Also, recent research has shown the biases of culture-independent techniques such as 16S rRNA amplicon sequencing in the deep characterisation of the microbiome, which can result in an underestimation of the microbial diversity, especially low-abundant species, which can go undetected [35].

Our findings show that both cultures performed in batch and bioreactors can not only support the growth of the most abundant families in faeces but also uncover a hidden diversity inside the faecal inoculum. Although present in low abundance, we found 36 new families in bioreactors compared to the ones reported in the GMRepo. It has been suggested that modifications promoting biofilm growth or introducing a more complex substrate environment might encourage a re-organisation of the microbial community and encourage the emergence of new functional niches. We observed differences in the detected ASVs between the lumenal and mucosal compartments of the bioreactors. For instance, in the study of M-ARCOL_Deschamps_et_al_2020, adding a mucosal component increased the number of newly detected ASVs compared to the faecal sample (Fig. 6B). Nevertheless, the abundance of the ASVs varied greatly, ranging from 500 to 5000. The significance of these changes needs further exploration, as it is necessary to rule out external contamination as the source of these new ASVs, especially considering their low abundance. Nonetheless, if a species of interest is enriched in the bioreactor, further enrichment protocols can promote its growth, isolation, and characterisation. This approach has proven successful in cultivating challenging microorganisms such as anammox bacteria at purities of up to 97% [15].

Although some of the families detected in the in vitro models were not found in GMRepo, a further literature search found documentation of *Gracilibacteraceae*, *Muribaculaceae*, *Defluviitaleaceae*, and *Vallitaleaceae* families identified in faecal microbiota samples [36–39]. Finally, the detection of the family *Vallitaleaceae* is surprising, as members of this family originated from hydrothermal fields and deep oceanic subsurface habitats rich in hydrocarbons [40]. Taken together, these results exhibit the potential of bioreactors to recover low abundant bacteria in faecal inocula and point to vast diversity yet to be cultured in these devices. The detailed list with the names of these families is available in Additional file 1: Table S3.

### Changes in the Microbial Diversity Inside Bioreactors

Regardless of the bioreactor model, once the microbial community is extracted from its natural environment, subsequent ecological changes occur inside this new artificial environment. Moreover, the culture of the faecal microbiota in the bioreactors is marked by a loss of diversity. For instance, in the study M-SHIME_Chassaing_et_al_2020, there was a decline of 50% in the α-diversity of the bioreactor after a stabilisation period of 7 days post-inoculation [41]. This loss of diversity might be explained by (1) the loss of species that cannot grow because their nutrient requirements rely on compounds provided by the host, (2) the loss of species whose nutrition relies on co-metabolism and depend on the presence of other species, and (3) the artificial selection of certain bacterial populations that use more effectively the available nutrients.

Alternatively, we propose another complementary explanation to account for the loss of diversity besides the death of multiple species [42]. We hypothesise that the selective pressure imposed by the conditions in the bioreactor can cause a decrease in the abundance (concentration of cells) of some species, which pushes them below the limit of detection of the sequencing method. In previous work, Gutierrez et al. [43] inoculated a soil sample in packed bioreactors fed with citrate as the sole carbon source and tracked the changes in the microbial community structure using 16S rRNA gene sequencing. Interestingly, some bacterial groups observed in the soil were not detected in the bioreactor. However, the bioreactor allowed the enrichment of new bacterial species undetected in the gene sequences of the soil sample. Then, they used the bioreactor’s effluent to fertilise sterile soil and found some bacterial species detected in the original inoculum, which apparently had disappeared in the bioreactor.

We suggest that the environmental conditions in the bioreactors, especially the absence of a human host, create new niche spaces that drive the replacement and proportional changes in the microbial community composition. These changes allow the growth of microorganisms whose activity is obscured by the presence of other, more dominant species in the human gastrointestinal tract. This feature highlights the potential of using bioreactors to enrich these microorganisms, allowing the role of these low-abundant species to be investigated.

Further research is required to understand the effect of the operational parameters of the bioreactor on diversity and to exploit the full potential of bioreactors to contribute new knowledge on gut microbial communities and to increase the number of species that can be cultured in vitro. We propose using bioreactors in tandem with culturomic approaches as another potential key to unlocking access to the microbial dark matter. More descriptive metadata of the environmental conditions of the communities growing in bioreactors is required to allow data from different studies to be co-modelled, which will accelerate our understanding of the value and limitations of bioreactors as a research tool. Bioreactors have proven to be powerful tools to enrich and even isolate species that are difficult to cultivate; therefore, they are emerging as a resource for studying microbial diversity and the ecological relationships of human microbiomes, especially those that inhabit the intestine. However, we must consider that each configuration and operation allows the enrichment of a group of microorganisms, overlapping or hiding others. Therefore, while the simulations of the intestine do not include all the environmental variables that the intestinal microorganisms experience, we must be cautious with drawing conclusions between the results obtained using in vitro models and a condition in human health.

## Supplementary Information

Below is the link to the electronic supplementary material.

Supplementary file1 (DOCX 1670 KB)

Supplementary file2 (CSV 7 KB)

## Data Availability

No new sequencing datasets were generated during the current study. Details of accession numbers for data analysed can be found in Additional file 1: Table S2. Original R scripts and metadata files are available in Zenodo ( https://zenodo.org/doi/10.5281/zenodo.8045625).
