# Supplementary material for: Meta-analysis of the Microbial Diversity Cultured in Bioreactors Simulating the Gut Microbiome
Source: Microb Ecol. 2024 Apr 8;87:57. doi: 10.1007/s00248-024-02369-0 (PMC11001690; doi:10.1007/s00248-024-02369-0)
Supplement: Supplementary file 1 [file 248_2024_2369_MOESM1_ESM.docx]

Meta-analysis of the microbial diversity cultured in bioreactors simulating the gut microbiome

Additional file 1

David Felipe García Mendez^1^, Siobhon Egan^1^, Julien Wist^1,3^, Elaine Holmes^1^, Janeth Sanabria^*1,2^

^1^Australian National Phenome Centre and Computational and Systems Medicine, Health Futures Institute, Murdoch University, Harry Perkins Building, Perth, Australia, WA 6150.

^2^Environmental Microbiology and Biotechnology Laboratory, Engineering School of Environmental & Natural Resources, Engineering Faculty, Universidad del Valle – Sede Meléndez, Cali, Colombia.

^3^Chemistry Department, Universidad del Valle, 76001, Cali, Colombia.

(*) Corresponding author

Fig. S1. Library size (total number of read counts) of the 1,512 samples from 18 projects included in the bioinformatic analysis.

Fig. S2. Rarefaction curves showing sequencing depth of the 1,512 samples across 19 projects after bioinformatic processing.

Fig. S3. Comparison of microbiome community structure from 19 different studies with 1,512 samples. Multi-dimensional scaling plot using Uniform Manifold Approximation and Projection (UMAP) on compositional data.

Fig. S4. Comparison of microbiome community structure from 19 different studies with 1,512 samples. (A) PCA scores plot for principal components PC1 and PC2; (B) PCA loadings plot for principal components PC1 and PC2; (C) Tridimensional plot depicting scores of the principal components PC1, PC2, and PC3. Please notice that the colours of the legend in plot (A) are the same ones used in the data points in plot (C).

Fig. S5. Comparison of microbiome community structure from 1,512 samples. Multi-dimensional scaling plot using t-distributed Stochastic Neighbor Embedding (t-SNE) with Hellinger distance measure on compositional data. (A) 16S rRNA region sequenced; (B) Country of donor used to inoculate bioreactor; (C) Presence of vitamins in the media.

Fig. S6. Comparison of microbiome community structure from 1,512 samples. Multi-dimensional scaling plot using t-distributed Stochastic Neighbor Embedding (t-SNE) with Hellinger distance measure on compositional data. (A) Number of stages in bioreactor system used; (B) Target gut component reproduced under in vitro conditions; (C) Predigestion step used in sample preparation; (D) Presence of vitamins in the media.

Fig. S7. Diversity profiles (Hill number at diversity orders q = 0-3) of the 19 selected studies (n = 1,512). Vertical bars show the standard error.

Fig. S8. Αlpha*-*diversity measures of samples (n = 1,512) included in the study with taxonomy collapsed to family level. (A) Observed, Chao1, Shannon and InvSimpson indexes grouped by study and ordered by bioreactor type along the x-axis. (B) Observed and Shannon indexes grouped by bioreactor type displaying significant pair-wise comparisons and (C) Shannon index grouped by donor category (age/health status of the donor) and type of input sample (pooled vs individual faecal samples) displaying significant pair-wise comparisons.

Fig. S9. Heatmap of top 10 bacteria Phyla identified in samples (remaining taxa grouped together in bottom row). Compositional data is represented as the total proportion of sequences from all samples in the project (n = 1,512).

Table S1. Dada2 parameters set up used during sequencing data processing

| SRA study identifier |  | Dada2 Parameter | | | | |
| --- | --- | --- | --- | --- | --- | --- |
|  | Sequence  length | truncLen | trimLeft | maxN | maxEE | truncQ |
| ERR021273 | 300 | 250 | 0 | 2 | 2 | 3 |
| ERP107582_forward | 150 | 150 | 0 | 2 | 2 | 2 |
| ERP107582_reverse | 150 | 150 | 0 | 2 | 2 | 2 |
| ERP108433_forward | 250 | 240 | 0 | 2 | 2 | 2 |
| ERP108433_reverse | 250 | 240 | 0 | 2 | 2 | 2 |
| ERP114897_forward | 250 | 240 | 0 | 2 | 2 | 2 |
| ERP114897_reverse | 250 | 240 | 0 | 2 | 2 | 2 |
| ERP120273_forward | 300 | 270 | 0 | 2 | 2 | 2 |
| ERP120273_reverse | 300 | 250 | 0 | 2 | 2 | 2 |
| ERP124577_forward | 300 | 250 | 0 | 2 | 2 | 2 |
| ERP124577_reverse | 300 | 210 | 0 | 2 | 2 | 2 |
| SRP164695_forward | 250 | 200 | 0 | 2 | 5 | 2 |
| SRP164695_reverse | 250 | 200 | 0 | 2 | 5 | 2 |
| SRP186729_forward | 300 | 200 | 0 | 2 | 5 | 2 |
| SRP186729_reverse | 300 | 190 | 0 | 2 | 5 | 2 |
| SRP247849_forward | 300 | 280 | 0 | 0 | 3 | 2 |
| SRP247849_reverse | 300 | 280 | 0 | 0 | 5 | 2 |
| SRP221446_forward | 250 | 245 | 0 | 0 | 4 | 2 |
| SRP221446_reverse | 250 | 245 | 0 | 0 | 4 | 2 |
| SRP241247_forward | 250 | 240 | 0 | 0 | 5 | 2 |
| SRP241247_reverse | 250 | 240 | 0 | 0 | 5 | 2 |
| SRP187837_forward | 300 | 300 | 0 | 0 | 3 | 2 |
| SRP187837_reverse | 300 | 270 | 0 | 0 | 5 | 2 |
| SRP263049_forward | 250 | 240 | 0 | 0 | 5 | 2 |
| SRP263049_reverse | 250 | 205 | 0 | 0 | 5 | 2 |
| SRP279236_forward | 250 | 240 | 0 | 0 | 5 | 2 |
| SRP279236_reverse | 250 | 205 | 0 | 0 | 5 | 2 |
| SRP287447_forward | 250 | 240 | 0 | 0 | 2 | 2 |
| SRP287447_reverse | 250 | 230 | 0 | 0 | 5 | 2 |
| SRP290868_forward | 250 | 240 | 0 | 0 | 5 | 2 |
| SRP290868_reverse | 250 | 240 | 0 | 0 | 5 | 2 |
| SRP307470 | 300 | 250 | 0 | 0 | 5 | 2 |
| SRP309452_forward | 250 | 250 | 0 | 0 | 3 | 2 |
| SRP309452_reverse | 250 | 210 | 0 | 0 | 5 | 2 |
| SRP320497_forward | 300 | 250 | 0 | 2 | 5 | 2 |
| SRP320497_reverse | 300 | 210 | 0 | 2 | 5 | 2 |
| SRP355931_forward | 300 | 250 | 0 | 0 | 5 | 2 |
| SRP355931_reverse | 300 | 250 | 0 | 0 | 5 | 2 |
| SRP369790_forward | 250 | 240 | 0 | 0 | 5 | 2 |
| SRP369790_reverse | 250 | 240 | 0 | 0 | 5 | 2 |
| SRP370478_forward | 250 | 240 | 0 | 0 | 4 | 2 |
| SRP370478_reverse | 250 | 210 | 0 | 0 | 5 | 2 |
| SRP373954_forward | 300 | 280 | 0 | 0 | 5 | 2 |
| SRP373954_reverse | 300 | 200 | 0 | 0 | 5 | 2 |
| SRP383204_forward | 250 | 245 | 0 | 0 | 4 | 2 |
| SRP383204_reverse | 250 | 240 | 0 | 0 | 5 | 2 |

Table S2. Selected studies and number of samples per project

| Model | Project id | Accession number | SRA study identifier | Total samples  in SRA  (n) | Samples selected for diversity analysis  (n) | Brief model description | Reference |
| --- | --- | --- | --- | --- | --- | --- | --- |
| Batch culture | batch_Bas-Bellver_et_al_2020 | PRJEB36995 | ERP120273 | 61 | 7 | 50 mL tubes/anaerobic gas chamber | [1] |
|  | batch_Elmen_et_al_2020 | PRJNA600537 | SRP241247 | 84 | 78 | Tubes/anaerobic gas chamber/shaking at 70 rpm | [2] |
|  | batch_Kim_et_al_2022 | PRJNA835228 | SRP383204 | 115 | 115 | Polypropylene tubes/anaerobic gas chamber | [3] |
|  | batch_Kleigrewe_et_al_2022 | PRJNA525885 | SRP187837 | 27 | 27 | 100 mL batch culture vessels/gas sparging/magnetic stirring/ automated pH controller | [4] |
|  | batch_Korth_et_al_2022 | PRJNA827123 | SRP370478 | 420 | 420 | 96-well plates/anaerobic gas chamber | [5] |
| ARCOL | ARCOL_Leclerc_et_al_2021 | PRJNA706948 | SRP309452 | 46 | 44(2)* | Single-stage bioreactor/computer controlled/microbiota generated anaerobiosis | [6] |
|  | M-ARCOL_Deschamps_et_al_2020 | PRJNA638256 | SRP266568 | 121 | 93(2)* | Single-stage bioreactor/computer controlled/microbiota generated anaerobiosis + addition of mucin beads | [7] |
| SHIME | SHIME_Firrman_et_al_2021 | PRJNA673917 | SRP290868 | 50 | 48(2)* | Included two TWIN-SHIME configurations:  Single colon: two-stage bioreactor. The first vessel mimics the stomach and small intestine digestion, and the second vessel mimics the ascending colon  3-stage colon: multi-stage bioreactor. Five vessels simulating the stomach, small intestine, ascending, transverse and descending colon | [8] |
|  | SHIME_Liu_et_al_2022 | PRJNA807003 | SRP359931 | 30 | 20 | Multi-stage bioreactor/ Five vessels simulating the stomach, small intestine, ascending, transverse and descending colon | [9] |
|  | SHIME_Ma_et_al_2022 | PRJNA825299 | SRP369790 | 45 | 43 | Multi-stage bioreactor/ Five vessels simulating the stomach, small intestine, ascending, transverse and descending colon | [10] |
|  | M-SHIME_Chassaing_et_al_2017 | PRJEB19279 | ERP021273 | 151 | 151 | Multi-stage bioreactor/ The set-up used in this study consisted of a stomach and a small intestine vessel and eight or nine proximal colon vessels in parallel + addition of carriers coated with agar containing porcin gastric mucin | [11] |
|  | M-SHIME_Abbeele_et_al_2021 | PRJNA701368 | SRP307470 | 75 | 75 | Multi-stage bioreactor/ Semi-continuous model for the parallel investigation of four different arms consisting of the two age groups and two treatments in a single setup consisting of 12 reactors (so-called QUAD-M-SHIME). Each arm consisted of a first reactor that simulated over time the stomach and small intestine and that operated according to a fill-and-draw principle. | [12] |
| SIMGI | SIMGI_Zorraquin_et_al_2021 | PRJNA731070 | SRP320497 | 6 | 3(2)* | Multi-stage bioreactor / Five interconnected Compartments that simulate the stomach, small intestine, ascending colon, transverse colon, and descending colon regions that can operate jointly or  independently.  In the present study, the system operated only with the stomach and small intestine compartments to simulate dynamic gastrointestinal digestion.  Faecal fermentations were performed in fermentation flasks (batch). | [13] |
| single-stage | single_stage_Xu_et_al_2019 | PRJNA604957 | SRP247849 | 108 | 72 | Single-stage colonic bioreactors/computer controlled/ gas sparging. | [14] |
| TIM-2 | TIM-2_Larsen_et_al_2019 | PRJEB25646 | ERP107582 | 132 | 132 | Model of proximal colon model/ tubular design, providing fully anaerobic conditions, peristaltic movements and removal of metabolites during fermentation | [15] |
|  | TIM-2_Vieira_et_al_2021 | PRJEB40878 | ERP124577 | 60 | 54 |  | [16] |
| Infors | Infors_Reese_et_al_2018 | PRJEB26446 | ERP108433 | 62 | 62 | Commercial bioreactor model manufactured by Infors | [17] |
| PolyFermS | PolyFermS_Pham_et_al_2019 | PRJEB32244 | ERP114897 | 117 | 68 | The fermentation setup consisted of a ﬁrst reactor with a working volume of 200 ml inoculated with 60 ml (30%, vol/vol) faecal beads from the respective donor (IR), which was connected to a control reactor (CR) and four test reactors (TRs). | [18] |
|  |  |  | Total | 1750 | 1512 |  |  |

* The number of faecal samples in these projects is indicated in brackets (). These were excluded during β- and α- diversity analysis but were used for the ASVs enrichment analysis as a reference.

Table S3. Comparison of the lists of families observed in the selected studies and the GMRepo database

| Families observed in the selected studies only | Families present in the GMRepo only |
| --- | --- |
| *Aggregatilineaceae*  *Ardenticatenaceae*  *Bryobacteraceae*  *Cerasicoccaceae*  *Chthoniobacteraceae*  *Cymatolegaceae*  *Defluviitaleaceae*  *Desulfitisporaceae*  *Desulfoplanaceae*  *Desulfosalsimonadaceae*  *Dethiobacteraceae*  *Dongiaceae*  *Eubacteriales XII. (i.s.)*  *Gracilibacteraceae*  *Haloplasmataceae*  *Immundisolibacteraceae*  *Kallotenuaceae*  *Ketobacteraceae*  *Lawsonellaceae*  *Micropepsaceae*  *Muribaculaceae*  *Nitrospinaceae*  *Oculatellaceae*  *Reyranellaceae*  *Spirillaceae*  *Syntrophorhabdaceae*  *Thermoanaerobaculaceae*  *Thermogemmatisporaceae*  *Thermohalobacteraceae*  *Thioalkalibacteraceae*  *Thiohalobacteraceae*  *Thiohalospiraceae*  *Trichocoleusaceae*  *Vallitaleaceae*  *Vermifilaceae*  *Vicinamibacteraceae* | *Acanthopleuribacteraceae*  *Acaryochloridaceae*  *Acetomicrobiaceae*  *Acidiferrobacteraceae*  *Acidilobaceae*  *Acidimicrobiaceae*  *Acidithiobacillaceae*  *Acidothermaceae*  *Actinopolymorphaceae*  *Actinopolysporaceae*  *Actinospicaceae*  *Afifellaceae*  *Ahrensiaceae*  *Alcanivoracaceae*  *Aminiphilaceae*  *Amoebophilaceae*  *Amorphaceae*  *Anaerotignaceae*  *Anaerovoracaceae*  *Anaplasmataceae*  *Ancalomicrobiaceae*  *Aphanizomenonaceae*  *Aphanothecaceae*  *Archaeoglobaceae*  *Arcobacteraceae*  *Arenicellaceae*  *Bacillales Family X. Incertae Sedis*  *Bacteriovoracaceae*  *Bartonellaceae*  *Beijerinckiaceae*  *Bernardetiaceae*  *Beutenbergiaceae*  *Blastochloridaceae*  *Blattabacteriaceae*  *Borreliaceae*  *Brachyspiraceae*  *Brevibacteriaceae*  *Brevinemataceae*  *Breznakiellaceae*  *Bruguierivoracaceae*  *Budviciaceae*  *Butyricicoccaceae*  *Caldicellulosiruptoraceae*  *Caldisericaceae*  *Caldisphaeraceae*  *Calditerrivibrionaceae*  *Calditrichaceae*  *Calotrichaceae*  *Caminicellaceae*  *Candidatus Babeliaceae*  *Candidatus Brocadiaceae*  *Candidatus Cloacimonadaceae*  *Candidatus Midichloriaceae*  *Catenulisporaceae*  *Celerinatantimonadaceae*  *Chamaesiphonaceae*  *Chelatococcaceae*  *Chitinibacteraceae*  *Chlamydiaceae*  *Chloroflexaceae*  *Chlorogloeopsidaceae*  *Chromobacteriaceae*  *Chroococcidiopsidaceae*  *Chrysiogenaceae*  *Chthonomonadaceae*  *Clostridiales Family XVI. Incertae Sedis*  *Coleofasciculaceae*  *Conexibacteraceae*  *Coraliomargaritaceae*  *Cryomorphaceae*  *Cryptosporangiaceae*  *Cuniculiplasmataceae*  *Cyanothecaceae*  *Cyclobacteriaceae*  *Deferribacteraceae*  *Dehalococcoidaceae*  *Deinococcaceae*  *Dermabacteraceae*  *Dermatophilaceae*  *Dermocarpellaceae*  *Desulfallaceae*  *Desulfarculaceae*  *Desulfatibacillaceae*  *Desulfatiglandaceae*  *Desulfatirhabdiaceae*  *Desulfitibacteraceae*  *Desulfobaccaceae*  *Desulfobacteraceae*  *Desulfobacteriaceae*  *Desulfococcaceae*  *Desulfofabaceae*  *Desulfolunaceae*  *Desulfonauticaceae*  *Desulfosarcinaceae*  *Desulfosudaceae*  *Desulfothermaceae*  *Desulfurellaceae*  *Desulfurococcaceae*  *Dictyoglomaceae*  *Dietziaceae*  *Echinimonadaceae*  *Elioraeaceae*  *Elusimicrobiaceae*  *Entomoplasmataceae*  *Fastidiosibacteraceae*  *Ferrimonadaceae*  *Ferroplasmaceae*  *Fibrobacteraceae*  *Filifactoraceae*  *Flammeovirgaceae*  *Flexibacteraceae*  *Flexistipitaceae*  *Fortieaceae*  *Francisellaceae*  *Frankiaceae*  *Fulvivirgaceae*  *Gallionellaceae*  *Geitlerinemataceae*  *Gemmatimonadaceae*  *Geoalkalibacteraceae*  *Geopsychrobacteraceae*  *Gloeobacteraceae*  *Glycomycetaceae*  *Gomontiellaceae*  *Gordoniaceae*  *Granulosicoccaceae*  *Hahellaceae*  *Haladaptataceae*  *Halanaerobiaceae*  *Haliscomenobacteraceae*  *Haloarculaceae*  *Halobacteriaceae*  *Halobacteriovoraceae*  *Halococcaceae*  *Haloferacaceae*  *Halorubraceae*  *Halothecacae*  *Halothermotrichaceae*  *Halothiobacillaceae*  *Hapalosiphonaceae*  *Herpetosiphonaceae*  *Holophagaceae*  *Hoyosellaceae*  *Hydrogenophilaceae*  *Hydrogenothermaceae*  *Hyellaceae*  *Idiomarinaceae*  *Ignatzschineriaceae*  *Ignavibacteriaceae*  *Isosphaeraceae*  *Jatrophihabitantaceae*  *Jiangellaceae*  *Jonesiaceae*  *Kaistiaceae*  *Kangiellaceae*  *Kopriimonadaceae*  *Kordiimonadaceae*  *Kosmotogaceae*  *Kribbellaceae*  *Ktedonobacteraceae*  *Kytococcaceae*  *Labilitrichaceae*  *Leeiaceae*  *Lentisphaeraceae*  *Leptospiraceae*  *Lewinellaceae*  *Litorivicinaceae*  *Magnetococcaceae*  *Magnetospirillaceae*  *Maricaulaceae*  *Marinifilaceae*  *Marinobacteraceae*  *Marivirgaceae*  *Melioribacteraceae*  *Merismopediaceae*  *Mesoaciditogaceae*  *Metamycoplasmataceae*  *Methanobacteriaceae*  *Methanocaldococcaceae*  *Methanocellaceae*  *Methanococcaceae*  *Methanocorpusculaceae*  *Methanomassiliicoccaceae*  *Methanomethylophilaceae*  *Methanomicrobiaceae*  *Methanoregulaceae*  *Methanosarcinaceae*  *Methanospirillaceae*  *Methanothermaceae*  *Methanotrichaceae*  *Methermicoccaceae*  *Methylacidiphilaceae*  *Microbulbiferaceae*  *Microcystaceae*  *Micromonosporaceae*  *Microscillaceae*  *Moorellaceae*  *Moritellaceae*  *Nakamurellaceae*  *Nannocystaceae*  *Natrialbaceae*  *Natronincolaceae*  *Natronoarchaeaceae*  *Neomegalonemataceae*  *Nevskiaceae*  *Nitratiruptoraceae*  *Nitriliruptoraceae*  *Nitrosopumilaceae*  *Nitrospiraceae*  *Nocardiopsaceae*  *Nodosilineaceae*  *Opitutaceae*  *Ornithinimicrobiaceae*  *Oscillatoriaceae*  *Owenweeksiaceae*  *Parachlamydiaceae*  *Parvibaculaceae*  *Patulibacteraceae*  *Perlucidibacaceae*  *Persicobacteraceae*  *Petrotogaceae*  *Phycisphaeraceae*  *Picrophilaceae*  *Pirellulaceae*  *Polyangiaceae*  *Porticoccaceae*  *Prochlorotrichaceae*  *Prolixibacteraceae*  *Promicromonosporaceae*  *Pseudanabaenaceae*  *Pseudohongiellaceae*  *Psychromonadaceae*  *Pyrodictiaceae*  *Rarobacteraceae*  *Reichenbachiellaceae*  *Rhodobiaceae*  *Rhodothalassiaceae*  *Rhodothermaceae*  *Rhodovibrionaceae*  *Rickettsiaceae*  *Rivulariaceae*  *Roseiflexaceae*  *Roseivirgaceae*  *Ruaniaceae*  *Rubrobacteraceae*  *Salinarimonadaceae*  *Salinibacteraceae*  *Salinisphaeraceae*  *Salisaetaceae*  *Sanguibacteraceae*  *Saprospiraceae*  *Scytonemataceae*  *Segniliparaceae*  *Sneathiellaceae*  *Solibacteraceae*  *Sphaerobacteraceae*  *Sphaerochaetaceae*  *Sphingosinicellaceae*  *Spirochaetaceae*  *Spirulinaceae*  *Spongiibacteraceae*  *Sporichthyaceae*  *Sporolactobacillaceae*  *Stappiaceae*  *Steroidobacteraceae*  *Stigonemataceae*  *Sulfolobaceae*  *Sulfuricellaceae*  *Sulfurimonadaceae*  *Sulfurospirillaceae*  *Sulfurovaceae*  *Syntrophaceae*  *Syntrophobacteraceae*  *Temperatibacteraceae*  *Tepidanaerobacteraceae*  *Terasakiellaceae*  *Thalassobaculaceae*  *Thermithiobacillaceae*  *Thermoanaerobacterales Family IV. Incertae Sedis*  *Thermococcaceae*  *Thermodesulfatatoraceae*  *Thermodesulfobacteriaceae*  *Thermodesulfobiaceae*  *Thermodesulfovibrionaceae*  *Thermofilaceae*  *Thermoflexibacteraceae*  *Thermoleophilaceae*  *Thermomicrobiaceae*  *Thermonemataceae*  *Thermoplasmataceae*  *Thermoproteaceae*  *Thermosynechococcaceae*  *Thermotogaceae*  *Thiobacillaceae*  *Thiofilaceae*  *Thiolinaceae*  *Thiotrichaceae*  *Thorselliaceae*  *Tindalliaceae*  *Tropherymataceae*  *Trueperaceae*  *Tsukamurellaceae*  *Vallicoccaceae*  *Vulgatibacteraceae*  *Waddliaceae*  *Wenzhouxiangellaceae*  *Xenococcaceae*  *Zavarziniaceae*  *Zooshikellaceae*  *Zymomonadaceae* |

Table S4. Alpha diversity statistics (supporting Fig. S7).

|  | Observed | | | Chao1 | | | Shannon | | | Simpson | | |
| --- | --- | --- | --- | --- | --- | --- | --- | --- | --- | --- | --- | --- |
|  | Mean | Med | SD | Mean | Med | SD | Mean | Med | SD | Mean | Med | SD |
| Panel A |  |  |  |  |  |  |  |  |  |  |  |  |
| batch_Bas-Bellver_et_al_2020 | 36.71 | 38 | 7.04 | 36.71 | 38 | 7.04 | 2.29 | 2.42 | 0.32 | 5.97 | 6.5 | 1.96 |
| batch_Elmen_et_al_2020 | 16.94 | 17 | 5.46 | 16.94 | 17 | 5.46 | 1.5 | 1.66 | 0.55 | 3.68 | 3.45 | 1.82 |
| batch_Kim_et_al_2022 | 36.52 | 35 | 4.62 | 36.52 | 35 | 4.62 | 2.27 | 2.26 | 0.16 | 7.03 | 6.68 | 1.37 |
| batch_Kleigrewe_et_al_2022 | 34.56 | 35 | 7.22 | 34.56 | 35 | 7.22 | 1.63 | 1.76 | 0.33 | 3.33 | 3.32 | 0.93 |
| batch_Korth_et_al_2022 | 27.4 | 26 | 4.95 | 27.4 | 26 | 4.95 | 2.06 | 2.1 | 0.18 | 5.3 | 5.57 | 1.01 |
| single_stage_Xu_et_al_ 2019 | 25.28 | 24.5 | 5.66 | 25.28 | 24.5 | 5.66 | 1.88 | 1.88 | 0.36 | 4.13 | 3.44 | 1.91 |
| SHIME_Firrman_et_al_2021 | 30.69 | 32 | 4.4 | 30.69 | 32 | 4.4 | 2.21 | 2.3 | 0.33 | 6.39 | 6.76 | 2.36 |
| SHIME_Liu_et_al_2022 | 26.85 | 27 | 3.07 | 26.85 | 27 | 3.07 | 2.05 | 2.04 | 0.14 | 5.45 | 5.21 | 1.05 |
| SHIME_Ma_et_al_2022 | 21.12 | 21 | 2.74 | 21.12 | 21 | 2.74 | 1.31 | 1.32 | 0.15 | 2.66 | 2.6 | 0.47 |
| M-SHIME_Abbeele_2021 | 15.89 | 16 | 4.05 | 15.89 | 16 | 4.05 | 1.25 | 1.35 | 0.45 | 2.8 | 2.73 | 1.06 |
| M-SHIME_Chassaing_et_al_2017 | 24.6 | 24 | 5.81 | 24.6 | 24 | 5.81 | 1.63 | 1.66 | 0.36 | 3.81 | 3.52 | 1.32 |
| SIMGI_Zorraquin_et_al_2021 | 20.33 | 20 | 2.52 | 20.33 | 20 | 2.52 | 1.94 | 1.97 | 0.07 | 4.9 | 5.33 | 0.79 |
| TIM-2_Larsen_et_al_2019 | 27.98 | 27 | 5.13 | 27.98 | 27 | 5.13 | 1.5 | 1.49 | 0.2 | 3.25 | 3.34 | 0.66 |
| TIM-2_Vieira_et_al_2021 | 9.46 | 9 | 1.8 | 9.46 | 9 | 1.8 | 1.37 | 1.42 | 0.29 | 3.22 | 3.27 | 1.06 |
| Infors_Reese_et_al_2018 | 20.73 | 20 | 4.06 | 20.73 | 20 | 4.06 | 1.67 | 1.75 | 0.36 | 3.74 | 3.59 | 1.12 |
| PolyFermS_Pham_et_al_2019 | 19.94 | 20 | 2.37 | 19.94 | 20 | 2.37 | 2.01 | 2.05 | 0.19 | 5.26 | 5.32 | 1.1 |
| ARCOL_Leclerc_et_al_2021 | 12.18 | 12 | 1.91 | 12.18 | 12 | 1.91 | 1.17 | 1.17 | 0.1 | 2.56 | 2.6 | 0.33 |
| M-ARCOL_Deschamps_et_al_2020 | 26.56 | 24 | 8.47 | 26.56 | 24 | 8.46 | 1.66 | 1.73 | 0.46 | 3.85 | 3.54 | 1.77 |
| Panel B |  |  |  |  |  |  |  |  |  |  |  |  |
| batch | 28.16 | 27 | 7.51 | 28.16 | 27 | 7.51 | 2.01 | 2.1 | 0.34 | 5.34 | 5.6 | 1.57 |
| single | 25.28 | 24.5 | 5.66 | 25.28 | 24.5 | 5.66 | 1.88 | 1.88 | 0.36 | 4.13 | 3.44 | 1.91 |
| SHIME | 23.22 | 23 | 6.71 | 23.22 | 23 | 6.71 | 1.61 | 1.59 | 0.48 | 3.9 | 3.39 | 1.84 |
| SIMGI | 20.33 | 20 | 2.52 | 20.33 | 20 | 2.52 | 1.94 | 1.97 | 0.07 | 4.9 | 5.33 | 0.79 |
| TIM-2 | 22.61 | 25 | 9.52 | 22.61 | 25 | 9.52 | 1.46 | 1.48 | 0.23 | 3.24 | 3.33 | 0.79 |
| Infors | 20.73 | 20 | 4.06 | 20.73 | 20 | 4.06 | 1.67 | 1.75 | 0.36 | 3.74 | 3.59 | 1.12 |
| PolyFermS | 19.94 | 20 | 2.37 | 19.94 | 20 | 2.37 | 2.01 | 2.05 | 0.19 | 5.26 | 5.32 | 1.1 |
| ARCOL | 21.94 | 21 | 9.75 | 21.95 | 21 | 9.75 | 1.5 | 1.41 | 0.45 | 3.43 | 2.84 | 1.59 |
| Panel C |  |  |  |  |  |  |  |  |  |  |  |  |
| BFInfant_Toddler | 15.89 | 16 | 4.05 | 15.89 | 16 | 4.05 | 1.25 | 1.35 | 0.45 | 2.8 | 2.73 | 1.06 |
| FFInfant | 19.94 | 20 | 2.37 | 19.94 | 20 | 2.37 | 2.01 | 2.05 | 0.19 | 5.26 | 5.32 | 1.1 |
| Healthy | 27 | 26 | 7.12 | 27 | 26 | 7.12 | 1.85 | 1.91 | 0.4 | 4.62 | 4.55 | 1.76 |
| Lean_obese | 9.46 | 9 | 1.8 | 9.46 | 9 | 1.8 | 1.37 | 1.42 | 0.29 | 3.22 | 3.27 | 1.06 |
| Vegetarians | 16.94 | 17 | 5.46 | 16.94 | 17 | 5.46 | 1.5 | 1.66 | 0.55 | 3.68 | 3.45 | 1.82 |
| Individual | 24.88 | 24 | 7.12 | 24.88 | 24 | 7.12 | 1.83 | 1.94 | 0.42 | 4.55 | 4.71 | 1.61 |
| Pooled | 25.21 | 25 | 9.67 | 25.21 | 25 | 9.67 | 1.7 | 1.63 | 0.46 | 4.27 | 3.55 | 2.05 |

Table S5. Selected samples for enrichment analysis

| Project | NCBI-SRA run id | Source | Donor | Replicate |
| --- | --- | --- | --- | --- |
| M-ARCOL_Deschamps | SRR11963015 | Stool | A | 0 |
|  | SRR11962989 | Single_stage/Luminal | A | 0 |
|  | SRR11962990 | Single_stage/Mucosal | A | 0 |
|  | SRR11962962 | Stool | B | 0 |
|  | SRR11963062 | Single_stage/Luminal | B | 0 |
|  | SRR11963063 | Single_stage/Mucosal | B | 0 |
| SHIME_Firman | SRR12968087 | Stool | A | 1 |
|  | SRR12968038 | Single_stage/Luminal | A | 1 |
|  | SRR12968039 | Single_stage/Luminal | A | 2 |
|  | SRR12968084 | Single_stage/Mucosal | A | 1 |
|  | SRR12968085 | Single_stage/Mucosal | A | 2 |
|  | SRR12968042 | Stool | B | 1 |
|  | SRR12968054 | Multi_stage/Luminal/Ascending colon | B | 1 |
|  | SRR12968056 | Multi_stage/Luminal/Ascending colon | B | 2 |
|  | SRR12968045 | Multi_stage/Luminal/Transverse colon | B | 1 |
|  | SRR12968047 | Multi_stage/Luminal/Transverse colon | B | 2 |
|  | SRR12968049 | Multi_stage/Luminal/Descending colon | B | 1 |
|  | SRR12968051 | Multi_stage/Luminal/Descending colon | B | 2 |
|  | SRR12968055 | Multi_stage/Mucosal/Ascending colon | B | 1 |
|  | SRR12968052 | Multi_stage/Mucosal/Ascending colon | B | 2 |
|  | SRR12968046 | Multi_stage/Mucosal/Transverse colon | B | 1 |
|  | SRR12968043 | Multi_stage/Mucosal/Transverse colon | B | 2 |
|  | SRR12968050 | Multi_stage/Mucosal/Descending colon | B | 1 |
|  | SRR12968048 | Multi_stage/Mucosal/Descending colon | B | 2 |
| ARCOL_Leclerc | SRR13863485 | Stool | A | 0 |
|  | SRR13863481 | Single_Stage/10uM_NO | A | 0 |
|  | SRR13863469 | Single_Stage/100uM_NO | A | 0 |
|  | SRR13863484 | Stool | B | 0 |
|  | SRR13863457 | Single_Stage/10uM_NO | B | 0 |
|  | SRR13863445 | Single_Stage/100uM_NO | B | 0 |
| SIMGI_Zorraquin | SRR14585829 | Stool | A | 0 |
|  | SRR14585827 | Stool/Control_48h | A | 0 |
|  | SRR14585826 | Stool+DW | A | 0 |
|  | SRR14585824 | Stool+DW_48h | A | 0 |
| SHIME_Liu | SRR18036853 | Single_stage_control_0h | A | 1 |
|  | SRR18036849 | Single_stage_control_0h | A | 2 |
|  | SRR18036844 | Single_stage_control_48h | A | 1 |
|  | SRR18036839 | Single_stage_control_48h | A | 2 |
|  | SRR18036845 | Single_stage_control_0h | B | 1 |
|  | SRR18036840 | Single_stage_control_0h | B | 2 |
|  | SRR18036830 | Single_stage_control_48h | B | 1 |
|  | SRR18036827 | Single_stage_control_48h | B | 2 |
|  | SRR18036836 | Single_stage_control_0h | C | 1 |
|  | SRR18036855 | Single_stage_control_0h | C | 2 |
|  | SRR18036842 | Single_stage_control_48h | C | 1 |
|  | SRR18036838 | Single_stage_control_48h | C | 2 |
|  | SRR18036831 | Single_stage_control_0h | D | 1 |
|  | SRR18036828 | Single_stage_control_0h | D | 2 |
|  | SRR18036829 | Single_stage_control_48h | D | 1 |
|  | SRR18036826 | Single_stage_control_48h | D | 2 |
|  | SRR18036852 | Single_stage_control_0h | F | 1 |
|  | SRR18036848 | Single_stage_control_0h | F | 2 |
|  | SRR18036841 | Single_stage_control_48h | F | 1 |
|  | SRR18036837 | Single_stage_control_48h | F | 2 |

Table S6. Analysis of enriched families inside the bioreactor for five selected projects.

| Project | NCBI-SRA run id | Source | Enriched families* | n reads** |
| --- | --- | --- | --- | --- |
| M-ARCOL_Deschamps | SRR11962989 | Single_stage/Luminal | Bacillaceae  Burkholderiaceae  Robiginitomaculaceae  Thermohalobacteraceae | 29  13  3  8 |
|  | SRR11962990 | Single_stage/Mucosal | Bacillaceae  Streptosporangiaceae | 28  2 |
|  | SRR11963062 | Single_stage/Luminal | Comamonadaceae  Desulfohalobiaceae  Enterococcaceae | 13  45  14 |
|  | SRR11963063 | Single_stage/Mucosal | Comamonadaceae  Coriobacteriaceae  Desulfohalobiaceae  Enterococcaceae  Pseudomonadaceae | 18  18  46  13  4 |
| SHIME_Firman | SRR12968038  SRR12968039 | Single_stage/Luminal | Akkermansiaceae Archangiaceae Comamonadaceae Desulfohalobiaceae Desulfovibrionaceae  Mycoplasmataceae Peptoniphilaceae Porphyromonadaceae  Prochlorococcaceae Rikenellaceae  Synergistaceae Xanthomonadaceae | 2+4  852+433  954+143  42+46  6+12  3187+1839  5171+2928  6+7  17+10  1948+1648  1539+762  66+66 |
|  | SRR12968084  SRR12968085 | Single_stage/Mucosal | Archangiaceae Desulfohalobiaceae Desulfovibrionaceae Erysipelotrichaceae Mycoplasmataceae  Oxalobacteraceae Peptoniphilaceae Porphyromonadaceae Prochlorococcaceae Rikenellaceae  Streptococcaceae Synergistaceae | 471+968  42+445  44+28  20+41  759+531  5+3  6035+4520  41+40  19+16  2137+2640  11+3  308+193 |
|  | SRR12968054  SRR12968056 | Multi_stage/Luminal/Ascending colon | Enterococcaceae  Desulfohalobiaceae  Enterobacteriaceae  Planococcaceae  Pseudomonadaceae | 101+36  259+123  2182+2272  117+25  498+605 |
|  | SRR12968045  SRR12968047 | Multi_stage/Luminal/Transverse colon | Archangiaceae  Enterococcaceae  Desulfohalbiaceae  Desulfovibrionaceae  Enterobacteriaceae  Leptotrichiaceae  Pseudomonadaceae  Synergistaceae | 4920+3181  82+130  469+81  105+66  923+286  2943+2678  814+203  4949+4014 |
|  | SRR12968049  SRR12968051 | Multi_stage/Luminal/Descending colon | Acidaminococcaceae  Archangiaceae  Bacillaceae  Enterococcaceae  Desulfohalobiaceae  Desulfovibrionaceae  Enterobacteriaceae  Leptotrichiaceae  Planococcaceae  Pseudomonadaceae  Synergystaceae | 52+90  2740+5402  27+46  66+22  128+195  32+84  282+578  1361+3463  84+39  410+854  2747+5500 |
|  | SRR12968055  SRR12968052 | Multi_stage/Mucosal/Ascending colon | Archangiaceae  Enterobacteriaceae  Planococcaceae | 1142+39  384+258  68+12 |
|  | SRR12968046  SRR12968043 | Multi_stage/Mucosal/Transverse colon | Acidaminococcaceae  Archagiaceae  Desulfovibrionaceae  Enterobacteriaceae  Leptotrichiaceae  Synergistaceae | 16+6  1916+1225  21+27  30+33  236+352  2389+1625 |
|  | SRR12968050  SRR12968048 | Multi_stage/Mucosal/Descending colon | Acidaminococcaceae  Archangiaceae  Caulobacteraceae  Desulfovibrionaceae  Enterobacteriaceae  Enterococcaceae  Leptotrichiaceae  Synergistaceae | 12+48  1236+1639  6+6  8+29  53+107  26+3  436+517  1596+2400 |
| ARCOL_Leclerc | SRR13863481 | Single_Stage/10uM_NO | Alcaligenaceae  Atopobiaceae  Desulfohalobiaceae  Peptoniphilaceae  Sutterellaceae | 13  2  33  4  20 |
|  | SRR13863469 | Single_Stage/100uM_NO | Peptoniphilaceae  Synergistaceae | 198  14 |
|  | SRR13863457 | Single_Stage/10uM_NO | Alcaligenaceae  Desulfohalobiaceae  Morganellaceae  Sutterellaceae | 8  30  19  18 |
|  | SRR13863445 | Single_Stage/100uM_NO | Coriobacteriaceae  Desulfohalobiaceae  Morganellaceae  Prevotellaceae  Tannerellaceae | 4  8  23  7  29 |
| SIMGI_Zorraquin | SRR14585827 | Stool/Control_48h | Barnesiellaceae  Desulfovibrionaceae  Enterobacteriaceae  Peptoniphiliaceae  Rikenellaceae  Saccharospirillaceae  Sutterellaceae  Synergistaceae | 2  3  7  6  2  3  2  429 |
|  | SRR14585824 | Stool+DW_48h | Acidaminococcaceae  Sphingomonadaceae  Synergistaceae  Veillonellaceae | 11  5  147  2 |
| SHIME_Liu | SRR18036839  SRR18036844 | Single_stage_control_48h | Enterobacteriaceae  Veillonellaceae | 232+111  3729+3817 |
|  | SRR18036830  SRR18036827 | Single_stage_control_48h | - | - |
|  | SRR18036842  SRR18036838 | Single_stage_control_48h | Enterobacteriaceae  Eubacteriales.XIII  Streptococcaceae | 65+388  69+276  36+130 |
|  | SRR18036829  SRR18036826 | Single_stage_control_48h | Enterobacteriaceae | 3250+4552 |
|  | SRR18036841  SRR18036837 | Single_stage_control_48h | Enterobacteriaceae  Enterococcaceae | 8205+7451  19+13 |

* A family was considered to be enriched when it was not detected in the faecal inoculum (n reads = 0), and appeared later in the bioreactor (n reads > 1).

** When replicates were available, the number of reads for each replicate in provided (separated with the symbol “+”).

Table S7. Composition at the phylum level of unique families observed in selected studies and GMRepo.

| Phylum | Families only observed in selected studies (%) | Families only observed in GMRepo (%) |
| --- | --- | --- |
| *Actinomycetota* | 2.8 | 12.6 |
| *Bacteroidota* | 2.8 | 6.8 |
| *Mycoplasmatota* | 2.8 | 0.65 |
| *Nitrospinota* | 2.8 | 0 |
| *Verrucomicrobiota* | 5.6 | 0.97 |
| *Acidobacteriota* | 8.3 | 0.97 |
| *Thermodesulfobacteriota* | 8.3 | 6.5 |
| *Chloroflexota* | 11.1 | 1.61 |
| *Cyanobacteriota* | 11.1 | 9.4 |
| *Bacillota* | 19.4 | 5.8 |
| *Pseudomonadota* | 25 | 24.2 |
| *Thermoproteota* | 0 | 2.3 |
| *Euryarchaeota* | 0 | 7.1 |
| *Other* | 0 | 21.1 |

The percentages were obtained by dividing the number of families within each phylum by the total number of families in each category, which are 36 and 310, respectively.

References

1.	Bas-Bellver C, Andrés C, Seguí L, et al (2020) Valorization of Persimmon and Blueberry Byproducts to Obtain Functional Powders: In Vitro Digestion and Fermentation by Gut Microbiota. J Agric Food Chem 68:8080–8090. https://doi.org/10.1021/acs.jafc.0c02088

2.	Elmén L, Zlamal JE, Scott DA, et al (2020) Dietary Emulsifier Sodium Stearoyl Lactylate Alters Gut Microbiota in vitro and Inhibits Bacterial Butyrate Producers. Front Microbiol 11:892. https://doi.org/10.3389/fmicb.2020.00892

3.	Kim EH-J, Wilson AJ, Motoi L, et al (2022) Chewing differences in consumers affect the digestion and colonic fermentation outcomes: in vitro studies. Food Funct 13:9355–9371. https://doi.org/10.1039/D1FO04364A

4.	Chung WSF, Walker AW, Louis P, et al (2016) Modulation of the human gut microbiota by dietary fibres occurs at the species level. BMC Biol 14:1–13. https://doi.org/10.1186/s12915-015-0224-3

5.	Korth N, Parsons L, Van Haute MJ, et al (2022) The Unique Seed Protein Composition of Quality Protein Popcorn Promotes Growth of Beneficial Bacteria From the Human Gut Microbiome. Front Microbiol 13:

6.	Leclerc M, Bedu-Ferrari C, Etienne-Mesmin L, et al (2021) Nitric Oxide Impacts Human Gut Microbiota Diversity and Functionalities. mSystems 6:e0055821. https://doi.org/10.1128/mSystems.00558-21

7.	Deschamps C, Fournier E, Uriot O, et al (2020) Comparative methods for fecal sample storage to preserve gut microbial structure and function in an in vitro model of the human colon. Appl Microbiol Biotechnol 104:10233–10247. https://doi.org/10.1007/s00253-020-10959-4

8.	Firrman J, Liu L, Mahalak K, et al (2021) Comparative analysis of the gut microbiota cultured in vitro using a single colon versus a 3-stage colon experimental design. Appl Microbiol Biotechnol 105:3353–3367. https://doi.org/10.1007/s00253-021-11241-x

9.	Liu L, Lu Y, Xu C, et al (2022) The Modulation of Chaihu Shugan Formula on Microbiota Composition in the Simulator of the Human Intestinal Microbial Ecosystem Technology Platform and its Influence on Gut Barrier and Intestinal Immunity in Caco-2/THP1-Blue^TM^ Cell Co-Culture Model. Front Pharmacol 13:

10.	Ma F, Luo Y, Liu Y, et al (2022) The disruption on gut microbiome of Decabromodiphenyl ethane exposure in the simulator of the human intestinal microbial ecosystem (SHIME). Toxicol Appl Pharmacol 452:116194. https://doi.org/10.1016/j.taap.2022.116194

11.	Chassaing B, Wiele TVD, Bodt JD, et al (2017) Dietary emulsifiers directly alter human microbiota composition and gene expression ex vivo potentiating intestinal inflammation. Gut 66:1414–1427. https://doi.org/10.1136/gutjnl-2016-313099

12.	Abbeele PV den, Sprenger N, Ghyselinck J, et al (2021) A comparison of the in vitro effects of 2’fucosyllactose and lactose on the composition and activity of gut microbiota from infants and toddlers. Nutrients 13:1–23. https://doi.org/10.3390/nu13030726

13.	Zorraquín-Peña I, Taladrid D, Tamargo A, et al (2021) Effects of Wine and Its Microbial-Derived Metabolites on Intestinal Permeability Using Simulated Gastrointestinal Digestion/Colonic Fermentation and Caco-2 Intestinal Cell Models. Microorganisms 9:1378. https://doi.org/10.3390/microorganisms9071378

14.	Xu Y, Chen Y, Xiang S, et al (2019) Effect of xylitol on gut microbiota in an in vitro colonic simulation. Turk J Biochem 44:646–653. https://doi.org/10.1515/tjb-2018-0328

15.	Larsen N, Bussolo de Souza C, Krych L, et al (2019) Potential of Pectins to Beneficially Modulate the Gut Microbiota Depends on Their Structural Properties. Front Microbiol 10:223. https://doi.org/10.3389/fmicb.2019.00223

16.	(2021) Impact of a fermented soy beverage supplemented with acerola by-product on the gut microbiota from lean and obese subjects using an in vitro model of the human colon. Appl Microbiol Biotechnol 105:3771–3785. https://doi.org/10.1007/s00253-021-11252-8

17.	Oliphant K, Parreira VR, Cochrane K, Allen-Vercoe E (2019) Drivers of human gut microbial community assembly: coadaptation, determinism and stochasticity. ISME J 13:3080–3092. https://doi.org/10.1038/s41396-019-0498-5

18.	Pham VT, Chassard C, Rifa E, et al (2019) Lactate Metabolism Is Strongly Modulated by Fecal Inoculum, pH, and Retention Time in PolyFermS Continuous Colonic Fermentation Models Mimicking Young Infant Proximal Colon. mSystems 4:1–22. https://doi.org/10.1128/msystems.00264-18
